# The Microscope and Microscopical Technology

**Published:** 1873-01

**Authors:** 


### Books Reviewed.

*The Microscope and Microscopical Technology. A Text Book for Physicians and Students.* By Heinrich Frey. Translated from the German and Edited by Geo. R. Cutter, M. D., New York: William Wood & Co., 1872. Buffalo: H. H. Otis.

The study of disease by the aid of the microscope has come to be one of the established methods employed at the present time, and it is an evidence of progress that physicians are using this method of investigating diseased action. By the student and practitioner commencing the study of microscopy there has been felt a want of a suitable text book to assist and guide them in their studies. Many of the works that have been issued have either been too narrow in scope or have treated of the subject in terms which to those commencing the study of the science, could not be understood.

The work of Dr. Frey, in the original German, has passed through four editions and those who were able to read the language it was highly prized for its simplicity and thoroughness. The wish for an English edition of the work has been often expressed by those who knew of its value but were unable to peruse its contents.

The work as now issued in English is divided into twenty-two sections, treating of the microscope and its use, the preparation of objects, the mounting and arranging of microscopic objects, etc,, etc.

Each subject receives full and ample treatment, and is written in language easily understood and appreciated by the beginner.

The directions for the use of the microscope are ample and should enable all after a little practice to become expert in its manipulation. The directions for investigating the various tissues and parts of the body in a normal and pathological state are clearly given and are sufficiently comprehensive in their scope to be of use to both the amateur and the expert microscopist. No words of ours will however convey to our readers an idea of the work so well as an attentive perusal by themselves, and we advise all commencing the study of microscopy to purchase Frey on the Microscope.

